# Detection of Bax Microsatellite Mutations and BaxΔ2 Isoform in Human Buccal Cells

**DOI:** 10.4172/2157-7013.S8-002

**Published:** 2015-07-17

**Authors:** Honghong Zhang, Cecilie Tassone, Nora Lin, Adriana Mañas, Yu Zhao, Jialing Xiang

**Affiliations:** Department of Biology, Illinois Institute of Technology, 3101 South Dearborn Street, Chicago, IL 60616

**Keywords:** Cheek cells, buccal cells, microsatellite mutation, microsatellite instability, Bax, BaxΔ2, chemotherapy

## Abstract

Loss of the pro-apoptotic Bcl-2 family protein Bax occurs in ~50% of hereditary nonpolyposis colorectal cancer (HNPCC) due to microsatellite instability (MSI). Recently, we found that some of the “Bax-negative” MSI tumor cells contain a functional Bax isoform, BaxΔ2, which sensitizes cells to selective chemotherapeutics. Here we show the detection of Bax microsatellite mutations and expression of BaxΔ2 proteins in human buccal cells. Our study provides a sensitive and non-invasive approach and a potential clinical application in diagnosis and treatment of MSI colon cancer patients.

## Letter to the Editor

Over 90% of hereditary nonpolyposis colorectal cancer (HNPCC, or Lynch syndrome) patients have high microsatellite instability (MSI-H) [1,2]. As HNPCC is caused by germline mutations inherited in an autosomal dominant pattern, the mutations can be detected not only in colon but also in other tissues such as blood and cheek cells [3,4]. In this study, we investigated a microsatellite mutation of tumor suppressor Bax gene in cheek cells. Deletion or insertion of a single guanine nucleotide (G) in the Bax exon 3 microsatellite track results in reading frameshift and premature termination in the Bax transcript, leading to a “Bax-negative” phenotype [5]. Recently, we found that some “Bax-negative” MSI tumor cells contain a functional Bax isoform, BaxΔ2, which is generated when a unique alternative splicing “salvages” the microsatellite frameshift mutation [6]. Therefore, BaxΔ2 can be produced in the Bax microsatellite-mutated cells. Interestingly, BaxΔ2 promotes cell death through the non-canonical mitochondrial pathway and sensitizes cancer cells to selective chemotherapeutics [7]. Currently, detection of MSI mutations relies primarily on analysis of clinical biopsy samples. The detection of Bax microsatellite mutations and BaxΔ2 protein expression in human cheek cells may provide a simple, sensitive and non-invasive screening for potential diagnosis and treatment of a subgroup of MSI colorectal cancer patients.

**Figure 1:**
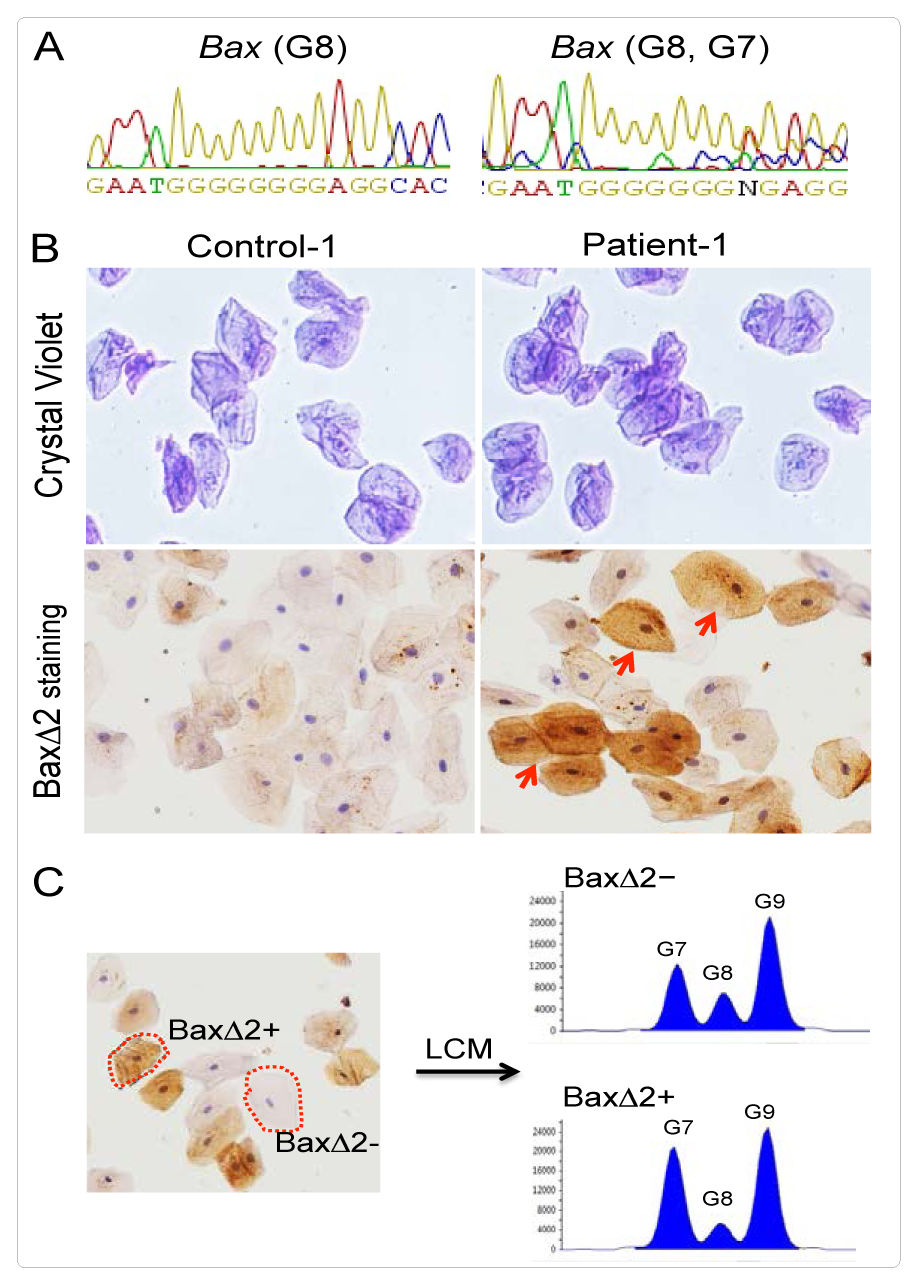
Detection of Bax microsatellite mutation and BaxΔ2 isoform protein expression in human buccal cells. (A) Genomic DNA sequence of buccal cell samples from Control-1 (left) and Patient-1 (right) individuals. (B) Top, Crystal Violet staining of buccal cells from Control-1 and Patient-1 individuals; bottom, duplicate slides were immunohistochemically (IHC) stained with anti-BaxΔ2 antibody (2D4) [6]. The arrows indicate positive BaxΔ2 stained cells. (C) BaxΔ2-positive (BaxΔ2+) and -negative (BaxΔ2-) cells were isolated from Patient-1 IHC stained slide using a Zeiss PALM Laser Capture Microdissection System (LCM). The LCM captured cells (n=15 for each group) were subjected to genomic DNA isolation and fluorescence PCR with Bax primers covering Bax exon 3. The peak profiles were presented here using Peak ScannerTM Software v1.0.

Human buccal cells were collected through mouth-wash and all experiments were performed with Institutional Review Board (IRB) approval. Cells were collected from two control-individuals with no cancer history (denoted Control-1 and Control-2) and another two individuals from a family with HNPCC history (Patient-1 and Patient-2). Genomic DNA was isolated from their buccal cells and Bax microsatellite status was determined by PCR with Bax specific primers and sequenced as described previously [6]. The representative sequence results from two individuals are shown in (Figure 1A) (Control-1, left panel; Patient-1, right panel). The homogenous wild type Bax microsatellite sequence containing eight guanines (G8) was detected in both control samples, while heterogeneous Bax (G7, G8) was detected in both patient samples, consistent with the corresponding individuals’ clinical history.

To analyze whether Bax (G7, G8) cells express BaxΔ2 protein, freshly isolated buccal cells from both control and patient samples were washed and spun onto glass slides by Cytospin centrifugation. The cell morphology was visualized by Crystal Violet staining (Figure 1B, top panel) and the duplicated slides were subjected to immunohistochemical (IHC) staining with anti-BaxΔ2 antibody, which has no cross-reactivity with Baxα [6]. Figure 1B (bottom panel) shows that strong BaxΔ2-positive staining was detected only in the Bax (G7, G8) sample but not in the Bax G8 sample. However, only about 35% of cells in the Bax (G7, G8) sample were BaxΔ2-positive. This suggested two possibilities. One was that there were mixed populations, i.e., some cells contained Bax G8/G8 alleles while others contained Bax G7/G7 alleles. Another possibility was that all cells contained Bax G7/G8 mixed alleles, but only a portion of them expressed BaxΔ2 proteins. To distinguish between these two possibilities, we isolated both BaxΔ2-positive and -negative cells from the Patient-1 sample slide using Laser Capture Microdissection (LCM) after IHC staining. About 15 cells were captured for each group. Genomic DNA samples were prepared from the captured cells and analyzed for Bax microsatellite status using fluorescence PCR. As shown in Figure 1C, the fluorescent peak profiles from both BaxΔ2-positive and -negative cells were similar. Both samples not only contain a mixture of Bax G7 and G8 but also G9. These results demonstrate that not all cells harboring Bax G7 mutations are able to generate BaxΔ2 proteins. Analysis of human buccal cells could provide a sensitive and non-invasive screening of Bax microsatellite status and BaxΔ2 protein expression with potential clinical applications in the treatment of MSI colon cancer patients.
